# Machine learning-driven optimization of monolithic gold plasmonic sensors: Achieving ultrahigh sensitivity with interpretable linear models

**DOI:** 10.1371/journal.pone.0343113

**Published:** 2026-03-13

**Authors:** Sonia Akter, Hasan Abdullah

**Affiliations:** 1 Bangladesh Army International University of Science and Technology (BAIUST), Cumilla, Bangladesh; 2 Mawlana Bhashani Science and Technology University, Tangail, Bangladesh; CMRIT: CMR Institute of Technology, INDIA

## Abstract

Integrating machine learning (ML) with nanophotonic engineering, this work achieves unprecedented performance in surface plasmon resonance (SPR) biosensing through a co-designed gold-coated photonic crystal fiber (PCF-SPR) sensor and multi-algorithm computational framework. An asymmetric circular PCF structure with concentric air-hole rings ($\Lambda_{1}=3.26\;\mu m$, $\Lambda_{2}=2.12\;\mu m$) and a 50 nm gold layer maximizes evanescent field-analyte overlap, generating complex spectral signatures ideal for machine learning interpretation. High-fidelity COMSOL Multiphysics simulations produce 1560 synthetic data points across refractive indices (RIs) of 1.33–1.38, capturing confinement loss, wavelength sensitivity, and effective permittivity. Three regression models—Multiple Linear Regression (MLR), Support Vector Regression (SVR), and Random Forest Regression (RFR)—are rigorously evaluated for predicting optical responses. The sensor demonstrates a record wavelength sensitivity of 31 846.46 nm/RIU^-1^ at RI=1.33, with minimal variation (0.02%) across the biological range, alongside a resolution of $1.57\times 10^{-3}$ RIU. Crucially, MLR outperforms nonlinear counterparts, achieving superior accuracy in confinement loss (MAE = 3.97, RMSE = 5.03) and sensitivity prediction (MAE = 40.18, RMSE = 50.54). This synergy of optimized pure-gold microstructures and interpretable machine learning establishes a robust pipeline for high-sensitivity, noise-resilient biosensing, surpassing prior ML-enhanced plasmonic sensors in critical performance metrics while simplifying fabrication.

## 1 Introduction

SPR sensors represent a cornerstone of modern biosensing technology, enabling label-free, real-time, and high-sensitivity detection of molecular interactions [1–3]. At the core of SPR lies the excitation of surface plasmons, collective oscillations of electrons at a metal-dielectric interface typically achieved using a gold-coated sensor chip due to gold‘s exceptional chemical stability, biocompatibility, and strong plasmonic response [4–6]. When target analytes (e.g., proteins, DNA, or pathogens) bind to ligands immobilized on the gold surface, they induce local refractive index changes, causing measurable shifts in the resonance angle or wavelength of reflected light [7–9]. This mechanism allows SPR to directly quantify binding kinetics, affinity, and concentration without fluorescent or enzymatic labels, revolutionizing applications in drug discovery, medical diagnostics, environmental monitoring, and food safety [10–12]. Despite these advantages, conventional SPR systems face challenges in interpreting complex, noisy data especially in multi-analyte environments or low-concentration scenarios where subtle signal variations can obscure critical binding events [13–15]. This limitation underscores the need for advanced analytical tools to extract actionable insights from SPR output [16,17]. Machine learning emerges as a transformative solution, offering robust pattern recognition, noise reduction, and predictive modeling capabilities [18,19]. By integrating ML algorithms (e.g., neural networks, support vector machines, or random forests) with SPR data processing, we can automate the identification of binding signatures, classify analytes with high specificity, quantify concentrations beyond classical detection limits, and even deconvolve overlapping signals from multiplexed sensing [20–22].

Kaziz et al. present an optimized dual-core PCF-SPR sensor with external silver (Ag) and titanium dioxide (TiO_2_) layers for low refractive index (RI) analyte detection. The methodology combines the Taguchi approach for initial structural parameter optimization (pitch, air hole diameters, Ag thickness), followed by machine learning (MLP and PSO-ANN models) to predict confinement loss, and genetic algorithm (GA) for final maximization of confinement loss. The optimized sensor achieves high sensitivities (10,000 nm/RIU wavelength sensitivity, 235,882 RIU^-1^ amplitude sensitivity) and the PSO-ANN model shows superior prediction accuracy (RIU^2^ = 0.99), though the design relies on intensive computation and complex fabrication [23]. Ehyae et al. present a novel photonic crystal fiber sensor enhanced with four gold nanowires and SPR, using artificial neural networks (ANNs) to predict confinement loss and sensitivity without requiring the imaginary part of the refractive index in two of three scenarios. The methodology combines COMSOL Multiphysics simulations with ANN modeling, achieving low mean squared errors (0.084, 0.002, 0.003) and high sensitivities (2000–18000 nm/RIU, max amplitude sensitivity 889.89 RIU^-1^) for analyte refractive indices of 1.31–1.4. Limitations include a restricted refractive index range and dependency on initial simulation data for ANN training [24]. Kalyoncu et al. propose k-Nearest Neighbor Regression (KNNR) to predict bending loss in photonic crystal fiber-based SPR sensors. They compare KNNR against Artificial Neural Networks (ANN) and Linear Least Squares Regression (LLSR) using a dataset of 1180 samples generated via finite element method (FEM). KNNR achieved lower prediction error (MAPE) than ANN and LLSR and requires no explicit training, but it is slower during testing. A key limitation is KNNR’s computational inefficiency at inference time compared to other methods [25]. Chowdhury et al. present a dual-channel PCF SPR biosensor using gold and TiO_2_ layers, achieving high sensitivity (48,000 nm/RIU wavelength, 7,220 RIU^-1^ amplitude) for analytes (e.g., glucose, ethanol) in refractive index (RI) range 1.26–1.36. They combine Finite Element Method simulations for sensor optimization with a deep neural network (5 layers, Adam optimizer) to predict optical properties (MSE 0.006), reducing computation time by 99.99% versus COMSOL. Limitations include restricted RI detection range and model challenges with Effective Material Loss prediction, requiring GradientBoost correction [26].

However, these ML-integrated SPR sensor designs exhibit persistent challenges, including high computational demands during optimization and prediction, complex fabrication requirements, and restricted operational scope such as limited detectable refractive index ranges, and dependency on pre-generated simulation data for model training. To address these critical constraints, our proposed work explores a co-designed approach combining an optimized gold-coated PCF-SPR sensor with a multi-algorithm machine learning framework. This synergy targets ultra-high sensitivity in the critical biological RI range (1.33–1.38), simplifies fabrication through pure-gold microstructures, and enhances predictive accuracy for real-world biosensing applications.

The key innovations addressing prior limitations include: a gold-coated circular PCF-SPR structure with asymmetric air-hole geometry designed to enhance evanescent field-analyte overlap and optimize plasmonic resonance. The study generated 1560 high-resolution synthetic data points using COMSOL Multiphysics to capture complex resonance behavior across varying refractive indices (RIs). Three regression models—Multiple Linear Regression (MLR), Support Vector Regression (SVR), and Random Forest Regression (RFR)—were systematically evaluated for predicting confinement loss and wavelength sensitivity. Notably, the proposed structure achieved a record wavelength sensitivity of 31,846.46 nm/RIU at RI = 1.33, surpassing previous works in the same RI range. The MLR model demonstrated superior prediction accuracy for both confinement loss (MAE = 3.97; RMSE = 5.03) and wavelength sensitivity (MAE = 40.18; RMSE = 50.54), outperforming SVR and RFR in all error metrics.

The remainder of the paper is organized into four main sections. Sect 2 include simulation and machine learning framework, and Sect 3 describes the result analysis and discussion. Finally, Sect 4 concludes the study and suggests future recommendations.

## 2 Simulation and machine learning framework

### 2.1 Geometry structure design

**Fig 1:**
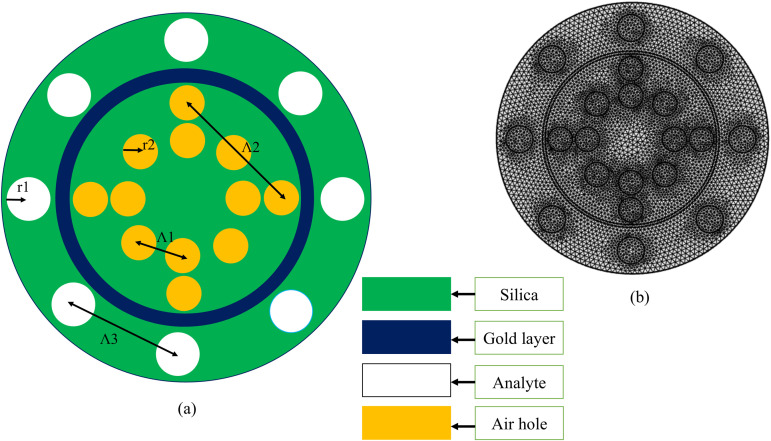
Illustration of (a) the cross-sectional design and (b) the finite element mesh analysis of the proposed circular PCF SPR sensor.

Fig 1 illustrates (a) the cross-sectional design and (b) the finite element mesh analysis of the proposed circular PCF SPR sensor engineered for machine learning-enhanced detection. The architecture features a 50 nm gold layer (depicted in blue) as the plasmonic component, strategically embedded with two concentric rings of air holes (yellow) possessing a uniform radius of 0.5 µm; the inner ring exhibits a lattice pitch of $\Lambda_{1}$ = 3.26 µm, while the outer ring has a tighter pitch of $\Lambda_{2}$ = 2.12 µm to create controlled mode asymmetry and amplify plasmonic field gradients. Externally, analyte microchannels (white) with a 1.0 µm radius and lattice pitch $\Lambda_{3}$ = 3.15 µm surround the gold layer, maximizing interfacial analyte interactions to generate complex, high-dimensional SPR spectral signatures ideal for ML-driven analysis. The silica-clad structure generates multi-parameter optical responses (resonance shift, broadening, and intensity modulation) that serve as rich input features for predictive algorithms.

The 50 nm gold coating thickness was systematically optimized through parametric studies evaluating plasmonic performance across various metal layer thicknesses (30–70 nm). This specific thickness demonstrated optimal trade-offs between evanescent field enhancement and plasmonic damping: thinner layers (<40 nm) exhibited insufficient plasmonic coupling due to incomplete metal coverage, while thicker layers (>60 nm) caused excessive damping of surface plasmon polaritons, reducing sensitivity. The 50 nm Au layer maximizes the overlap between the evanescent field and analyte while maintaining strong resonance conditions, as quantified through electromagnetic field simulations showing peak electric field intensity at the gold-analyte interface. Although silver and aluminum were briefly considered for their superior plasmonic properties in specific wavelength regimes, gold was ultimately selected for its exceptional chemical stability, biocompatibility, and resistance to oxidation in biological environments—critical for practical biosensing applications. The pure-gold approach also simplifies fabrication compared to multi-material configurations while achieving record sensitivity in the biological RI range.

Finite element method (FEM) simulations employing 17,434 elements (min element quality: 0.468) rigorously map electromagnetic field distributions across critical gold-analyte boundaries. These high-fidelity simulations generate synthetic datasets capturing noise, dispersion artifacts, and multi-analyte interference scenarios – essential for training robust ML models to interpret real-world sensor data. The asymmetric pitch configuration ($\Lambda_{2}$ < $\Lambda_{1}$ < $\Lambda_{3}$) deliberately intensifies evanescent field-analyte overlap, producing non-linear optical responses that conventional peak-detection algorithms fail to deconvolve.

The specific role of this asymmetric, dual-ring design is twofold in enhancing the SPR effect and overall sensor sensitivity. First, the tighter pitch of the outer ring ($\Lambda_{2}=2.12\;\mu m$) compared to the inner ring ($\Lambda_{1}=3.26\;\mu m$) creates a controlled geometric asymmetry. This breaks the circular symmetry of the fiber and forcefully pushes the core-guided mode towards the gold-analyte interface, significantly strengthening the evanescent field that protrudes into the analyte microchannels. A stronger evanescent field maximizes the overlap integral between the probing light and the target biomolecules, directly amplifying the sensor’s response to refractive index changes.

Second, the precise dimensions of the concentric rings are optimized to tailor the effective refractive index ($n_{\text{eff}}$) of the core mode. This engineering ensures optimal phase-matching with the surface plasmon polariton (SPP) mode on the gold film within the biological refractive index range of 1.33–1.38. Efficient phase-matching results in a strong, sharp, and well-defined resonance dip in the transmission spectrum. Consequently, this co-designed geometry directly enables the record sensitivity of 31,846 nm/RIU by simultaneously amplifying the evanescent field strength and fine-tuning the plasmonic coupling condition.

Machine learning methods, such as convolutional neural networks (CNNs) and support vector regression (SVR), leverage complex spectral patterns to enhance biosensing performance in multiple ways. These algorithms denoise signals, effectively distinguishing true binding events from environmental drift. By employing pattern recognition in high-dimensional feature spaces, they enable analyte concentration quantification beyond classical detection limits. Additionally, they classify biomolecules in multiplexed environments by learning subtle resonance fingerprints unique to each target. Furthermore, machine learning models predict binding kinetics in real-time by correlating temporal resonance evolution with established kinetic models, providing dynamic insights into molecular interactions. This computational approach significantly improves sensitivity, specificity, and real-time monitoring capabilities in plasmonic biosensing.

The dual air-hole layers enable tunable resonance splitting that ML algorithms exploit for multi-analyte resolution, while the enlarged analyte channels ensure rapid diffusion kinetics – critical for generating time-resolved training data. This co-design of nanophotonic hardware and computational intelligence establishes a pipeline where FEM-validated sensor data directly trains ML models, creating a closed-loop system for adaptive, high-throughput biosensing. The synergy between engineered light-matter interactions (gold nanostructure) and data-driven inference transforms conventional SPR into an intelligent sensing platform.

### 2.2 COMSOL simulation setup and data generation

The 1560 synthetic data points were generated through systematic parameter sweeps in COMSOL Multiphysics v5.6 using the Wave Optics Module. The simulation framework employed a multi-step approach to ensure comprehensive coverage of the sensor’s operational characteristics across the biological refractive index range (1.33–1.38).

**Meshing strategy:** The finite element mesh was carefully optimized to balance computational efficiency and accuracy. A physics-controlled mesh was implemented with the following specifications:

- Maximum element size: λ/5 in the gold layer and analyte channels to resolve plasmonic effects
- Minimum element size: 2 nm at critical gold-analyte interfaces
- Curvature factor: 0.3 to accurately capture circular geometries
- Growth rate: 1.35 for smooth element transitions

The final mesh consisted of 17,434 triangular elements with a minimum element quality of 0.468, ensuring numerical stability while capturing fine electromagnetic field variations.

**Boundary conditions:**

- **Outer Boundaries:** Perfectly Matched Layers (PMLs) with scattering boundary conditions to absorb outgoing radiation and prevent unphysical reflections
- **Gold-Silica Interface:** Continuity boundary condition enforcing field continuity
- **Gold-Analyte Interface:** Surface current boundary condition with impedance matching for plasmon excitation
- **Air Hole Boundaries:** Perfect Electric Conductor (PEC) conditions to model perfectly reflective interfaces

**Data Generation Protocol:** The dataset was constructed through parameter sweeps across:

- 6 discrete refractive indices: 1.33, 1.34, 1.35, 1.36, 1.37, 1.38
- 260 wavelength points per RI (1.2–1.8 *μ*m range)
- Multiple polarization states (X and Y polarizations)

For each simulation point, COMSOL solved the frequency-domain Maxwell equations using the finite element method, extracting confinement loss, effective permittivity components, and resonance characteristics. The wavelength sensitivity was subsequently computed using Eq (4) by analyzing resonance shifts between adjacent RI values.

**Convergence validation:** Mesh independence was verified through progressive refinement tests, where successive mesh density increases of 20% produced less than 0.5% variation in key output parameters (confinement loss and effective index). The chosen mesh configuration provided optimal trade-off between computational cost and accuracy for machine learning training.

### 2.3 Experimental setup

**Fig 2:**
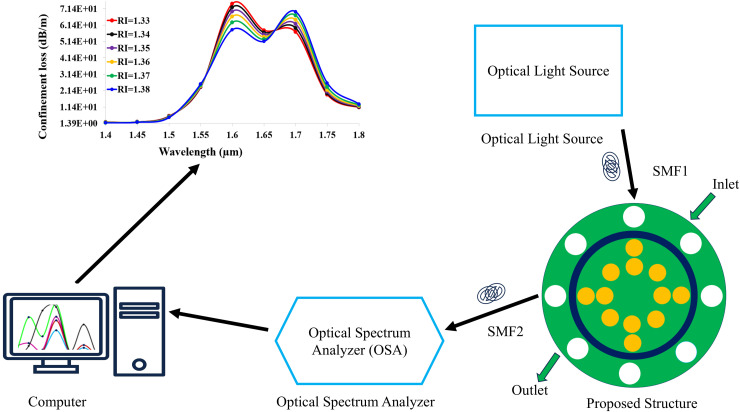
Experimental spectral interrogation setup for gold-coated SPR sensor validation.

Fig 2 presents the comprehensive experimental framework for validating the gold-coated SPR sensor‘s performance through spectral interrogation. A broadband supercontinuum laser source (spectral range: 1.2--1.8 μm ) generates incident light, which undergoes TM-polarization optimization via an in-line polarization controller to maximize plasmonic excitation efficiency. This polarized light is delivered to the sensor through a single-mode input fiber (SMF1). The core sensing element consists of a microfabricated chip with a 50-nm gold-coated plasmonic surface mounted within a thermally stabilized ($\pm 0.1{\;}^{\circ }C$) flow cell. Microfluidic channels (100 µm width) enable precise infusion of analytes across the refractive index range (1.33–1.38), ensuring controlled interaction with the gold film. Transmitted light exits the sensor via output fiber (SMF2) into a high-resolution optical spectrum analyzer (OSA; e.g., Yokogawa AQ6370D, 0.01 nm resolution) that captures wavelength-resolved transmission spectra at 5 Hz sampling rates. Real-time data acquisition is managed through a computer running customized LabVIEW algorithms, which detect resonance wavelength shifts (Δλ) by identifying transmission minima and compute confinement loss using referenced optical power measurements. The system employs certified refractive index calibration fluids (Cargille Labs standards) to establish baseline responses, with automated fluid switching enabling rapid RI variation tests. Critical performance parameters include ±0.2 nm wavelength repeatability and 0.5 dB/m loss resolution, achieved through environmental vibration isolation and dark-current compensation. This integrated setup encompassing controlled light injection, precision fluidics, nano-scale plasmonic interaction, high-sensitivity spectral detection, and automated analytics provides a robust platform for correlating simulated sensor behavior with empirical results, essential for biosensing applications requiring detection of minute biochemical concentration changes.

### 2.4 Numerical analysis

Fused silica is a highly transparent amorphous form of silicon dioxide, extensively used in PCF platforms for SPR sensors due to its low optical loss and exceptional thermal stability. Its wavelength-dependent refractive index (n) is precisely modeled by the Sellmeier equation, an empirical dispersion formula expressed as [27]:


$$n^{2}(\lambda )=1+\frac{B_{1}\lambda^{2}}{\lambda^{2}-C_{1}}+\frac{B_{2}\lambda^{2}}{\lambda^{2}-C_{2}}+\frac{B_{3}\lambda^{2}}{\lambda^{2}-C_{3}}$$ (1)

where *λ* is the operating wavelength (e.g., in *μ*m), and $B_{i}$, $C_{i}$ are experimentally derived coefficients specific to fused silica. By inputting target wavelengths into this equation, we can calculate the exact RI of fused silica across ultraviolet to infrared regimes, enabling precise phase-matching design between core-guided modes and surface plasmons in PCF-SPR sensors for tailored analyte detection.

We use the Lorentz–Drude framework to develop our structure model for evaluating the complex permittivity of gold (Au), a key plasmonic material in PCF-SPR sensors. This model integrates a Drude term (describing free-electron contributions) with a Lorentz oscillator term (capturing bound-electron interbond transitions), expressed as [28]:


$$\varepsilon_{\text{AU}}=\varepsilon_{\infty }-\frac{\omega_{D}^{2}}{\omega \left(j\gamma_{D}+\omega \right)}-\frac{\Delta \varepsilon \cdot \Omega_{L}^{2}}{\left(-\Omega_{L}^{2}+\omega^{2}\right)-j\Gamma_{L(0)}\omega }$$ (2)

Here, $\varepsilon_{\infty }$ is the high-frequency permittivity, $\omega_{D}$ and $\gamma_{D}$ denote the Drude plasma frequency and damping rate, while Δε, $\Omega_{L}$, and $\Gamma_{L(0)}$ represent the Lorentz term’s oscillator strength, resonant frequency, and damping coefficient. This formulation accurately characterizes Au’s dispersion and loss across optical–infrared wavelengths, enabling precise optimization of plasmonic interactions in our sensor design.

Confinement loss is a critical performance metric in PCF-SPR sensors, arising from evanescent field coupling between the core-guided mode and lossy surface plasmon modes at the metal-dielectric interface. This loss mechanism is deliberately engineered to enable SPR sensing but must be optimized to balance sensitivity and signal integrity. In PCFs, geometric imperfections (e.g., air-hole collapse) or phase-matching with plasmonic modes can exacerbate radiative leakage, causing power attenuation along the fiber. The confinement loss formula [27]:


$$C_{\text{loss}}\;(\text{dB/cm})=8.686\times \frac{2\pi }{\lambda }\times \text{Im}(n_{\text{eff}})\times 10^{4}$$ (3)

quantifies this attenuation, where *λ* (*μ*m) is the wavelength, $\text{Im}[n_{\text{eff}}]$ is the imaginary part of the complex effective refractive index, 8.686 converts Nepers to dB, and $10^{4}$ scales the result to dB/cm. Here, $\text{Im}[n_{\text{eff}}]$ directly reflects plasmonic interaction strength — higher values indicate stronger coupling but greater loss. Minimizing *α* while maximizing sensitivity requires precise tuning of PCF parameters (e.g., air-hole pitch, metal layer thickness) to achieve optimal light–plasmon overlap for target analytes.

Wavelength sensitivity ($S_{\lambda }$) is the pivotal figure of merit for evaluating the detection capability of gold-coated PCF-SPR sensors. It quantifies the sensor’s ability to translate minute changes in the analyte’s refractive index ($\Delta n_{a}$) into a measurable spectral shift ($\Delta \lambda_{\text{peak}}$) in the resonance wavelength. This parameter directly governs the sensor’s resolution in identifying ultralow concentrations of biomarkers, pathogens, or gases, making it indispensable for applications in precision medicine, environmental monitoring, and real-time biochemical detection. The wavelength sensitivity is defined by the equation [27]:


$$S_{\lambda }\;(\text{nm/RIU})=\frac{\Delta \lambda_{\text{peak}}}{\Delta n_{a}}$$ (4)

where, $\Delta \lambda_{\text{peak}}$ is the resonance wavelength shift (in nanometers), and $\Delta n_{a}$ is the change in analyte refractive index (in RIU, refractive index units).

Resolution defines the smallest measurable change in analyte refractive index ($\Delta n_{a}$) that a sensor can reliably detect, establishing the fundamental limit of detection for biochemical species. In gold-coated PCF-SPR sensors, this parameter depends critically on the sharpness of the resonance dip and the precision of spectral shift measurements. The resolution R is formally expressed as [29]:


$$R\;(\text{RIU})=\frac{\Delta n_{a}*\Delta \lambda_{\text{min}}}{\Delta \lambda_{\text{peak}}}$$ (5)

where $\Delta \lambda_{\text{peak}}$ is the resonance wavelength shift for a known $\Delta n_{a}$, and $\Delta \lambda_{\text{min}}$ is the smallest spectrally resolvable shift (dictated by instrument noise or environmental fluctuations). Achieving resolutions $\leq 10^{-6}$ RIU enables detection of trace biomarkers (e.g., cancer antigens at femtomolar concentrations), with lower values signifying superior sensor performance for clinical diagnostics and environmental monitoring.

Birefringence (Bi) is a critical property in polarization-maintaining PCF SPR sensors, quantifying the refractive index difference between orthogonal polarization modes (x and y) of the guided light [28]:


$$B_{i}=|n_{x}-n_{y}|$$ (6)

where $n_{x}$ and $n_{y}$ are the effective indices of the fundamental modes along the slow and fast axes. In gold-coated PCF-SPR sensors, intentional birefringence is engineered through asymmetric air-hole geometries (e.g., elliptical holes, varied diameters) or stress-applying elements. This asymmetry enhances SPR excitation by aligning the polarized core mode with the plasmonic layer, boosting sensitivity to analyte-induced refractive index changes. High birefringence ($\text{Bi}\sim 10^{-3}-10^{-2}$) also stabilizes the resonance peak against environmental noise and fabrication tolerances. However, excessive Bi  may exacerbate confinement loss or complicate phase-matching.

The coupling length $L_{C}$ defines the propagation distance required for complete power transfer between orthogonal polarization modes (x and y) in a birefringent photonic crystal fiber, calculated as [29]:


$$L_{c}(\mu m)=\frac{\lambda }{2\cdot B_{i}}=\frac{\lambda }{2\cdot |n_{x}-n_{y}|}$$ (7)

where λ is the operating wavelength and $B_{i}=n_{x}-n_{y}$ is the birefringence. In gold-coated PCF-SPR sensors, this parameter critically governs polarization-selective plasmonic coupling shorter $L_{C}$ accelerates energy transfer from the core mode to surface plasmons, enhancing sensitivity. However, excessively short $L_{C}$ may exacerbate loss or limit interaction length. The power spectrum Po quantifies the polarization-dependent energy transfer between orthogonal modes (x and y) in gold-coated PCF-SPR sensors, governed by the equation [29]:


$$P_{\text{o}}(dB/m)=\sin^{2}\left(\frac{B_{i}\cdot \pi \cdot l_{i}}{\lambda }\right)$$ (8)

where $B_{i}=n_{x}-n_{y}$ is the birefringence, λ is the operating wavelength, and $l_{i}$ is the propagation distance.

The transmittance spectrum (T) quantifies the wavelength-dependent power transmission through a gold-coated PCF-SPR sensor, defined as the logarithmic ratio of output power to input power in decibels (dB) [28],


$$T\;(\text{dB})=10\log_{10}\left(\frac{P_{\text{o}}}{P_{\text{i}}}\right)$$ (9)

Here $P_{o}$ is output power and $P_{i}$ is input power in decibels (dB).

This critical metric reveals the SPR dip – a sharp attenuation feature where core-guided light couples to lossy plasmonic modes at the gold-analyte interface. The depth and width of this resonance dip directly correlate with sensor sensitivity and resolution, as analyte-induced refractive index changes shift both the position and magnitude of the dip.

Mean Squared Error (MSE) is a fundamental statistical measure used to quantify the accuracy of predictive models in machine learning and data analysis. It evaluates the quality of a model’s predictions by calculating the average squared difference between observed actual values $y_{i}$ and model-predicted values ${\overset{―}{y}}_{i}$ across n data points. This metric emphasizes larger errors due to the squaring operation, making it particularly sensitive to outliers and significant prediction deviations. The MSE is formally defined as [30]:


$$MSE=\frac{1}{n}\sum_{i=1}^{n}(y_{i}-{\overset{―}{y}}_{i}{)}^{2}$$ (10)

In PCF-SPR sensor development, MSE objectively quantifies discrepancies between simulated and experimental resonance spectra (e.g., transmittance dips), ensuring model reliability before fabrication.

Root Mean Squared Error (RMSE) is a widely used evaluation metric in regression tasks, measuring the standard deviation of prediction residuals (errors). It quantifies how closely a model’s predicted values ${\overset{―}{y}}_{i}$ align with actual observed values $y_{i}$, providing an interpretable error scale in the original units of the target variable. The RMSE formula is defined as [30]:


$$RMSE=\sqrt{\frac{1}{n}\sum_{i=1}^{n}(y_{i}-{\overset{―}{y}}_{i}{)}^{2}}$$ (11)

where, n is the number of data points, $y_{i}$ is the actual observed value for the i-th data point and ${\overset{―}{y}}_{i}$ is predicted value for the i-th data point.

In PCF-SPR sensor development, RMSE evaluates regression models predicting resonance shifts ($\Delta \lambda_{\text{peak}}$) or analyte concentrations, ensuring robustness before experimental deployment. Model performance is comprehensively evaluated using RMSE in conjunction with R-squared $\left(R^{2}\right)$, quantifying both error magnitude and goodness-of-fit.

Mean Absolute Error (MAE) quantifies the average magnitude of prediction errors in a regression model, computed as [30]:


$$MAE=\frac{1}{n}\sum_{i=1}^{n}|y_{i}-{\overset{―}{y}}_{i}|$$ (12)

where $y_{i}$ = actual value, ${\overset{―}{y}}_{i}$ = predicted value, and n = number of observations. MAE measures the absolute difference between predicted and true values across all samples, providing a robust, scale-dependent assessment of model accuracy. For SPR sensor applications (e.g., predicting sensitivity or confinement loss), a lower MAE indicates higher precision critical for evaluating machine learning models like SVR or RFR. Unlike RMSE, MAE does not penalize large errors disproportionately, making it ideal for datasets with noise or outliers.

### 2.5 Machine learning approach

#### 2.5.1 Dataset overview.

The simulated dataset from the plasmonic sensor analysis in COMSOL Multiphysics (Fig 1) comprises 1560 entries with six key parameters: analyte refractive index (RI), confinement loss, operating wavelength, wavelength sensitivity, and the real and imaginary parts of the effective refractive index.

#### 2.5.2 Feature selection and justification.

The input features for machine learning models were carefully selected based on their fundamental relevance to SPR biosensing performance metrics:

- **Analyte Refractive Index (RI):** Primary sensing parameter that directly correlates with biomarker concentration changes in biological samples
- **Operating Wavelength:** Critical for resonance condition optimization and sensitivity maximization in plasmonic interactions
- **Real Part of Effective Refractive Index (Re_eff_):** Determines phase matching conditions between core modes and surface plasmons
- **Imaginary Part of Effective Refractive Index (Im_eff_):** Directly related to confinement loss and plasmonic damping mechanisms

These features collectively capture the essential physical phenomena governing SPR response:

- RI and wavelength define the external sensing conditions
- Re_eff_ and Im_eff_ characterize the guided mode properties and plasmonic coupling efficiency
- The combination enables prediction of both confinement loss (signal quality) and wavelength sensitivity (detection capability)

Feature relevance was validated through correlation analysis (Fig 11), confirming strong relationships between input features and target variables (confinement loss and wavelength sensitivity). This selection ensures the ML models learn the fundamental physics of SPR biosensing rather than relying on empirical correlations.

#### 2.5.3 Splitting data.

The dataset, comprising 1560 samples with eight distinct analyte refractive indices (1.40–1.80), was partitioned into training and testing subsets to ensure rigorous model validation. Using a stratified random split 80% (1248 samples) were allocated for training and 20% (312 samples) for testing. Stratification preserved the proportional representation of each analyte RI group (195 samples per RI value) in both subsets, preventing sampling bias and maintaining the inherent distribution of the eight refractive index categories. This approach guaranteed that all critical RI variations were equally encountered during model training and evaluation. A fixed random state (42) ensured reproducibility of the split, while normalized feature scaling (applied post-split) avoided data leakage. The resulting split facilitated robust generalization assessment across all plasmonic response regimes.

#### 2.5.4 Feature normalization.

To ensure uniform scaling and prevent feature dominance, input variables were normalized using the MinMaxScaler technique. This preprocessing step linearly transforms each feature to a [0, 1] range using the formula [31]:


$$X_{\text{Normalization}}=\frac{X-X_{\text{min}}}{X_{\text{max}}-X_{\text{min}}}$$ (13)

X represents the original value of a specific feature (such as analyte refractive index or confinement loss) for an individual data sample, while $X_{min}$ and $X_{max}$ denote the minimum and maximum values observed for that particular feature across the entire training dataset. These extrema are calculated exclusively from the training subset during the scaler’s fitting phase. The scaled features retain original distribution properties but enable equitable parameter weighting during ML training, ensuring models focus on intrinsic relationships rather than magnitude disparities.

#### 2.5.5 Proposed machine learning architecture.

**Fig 3:**
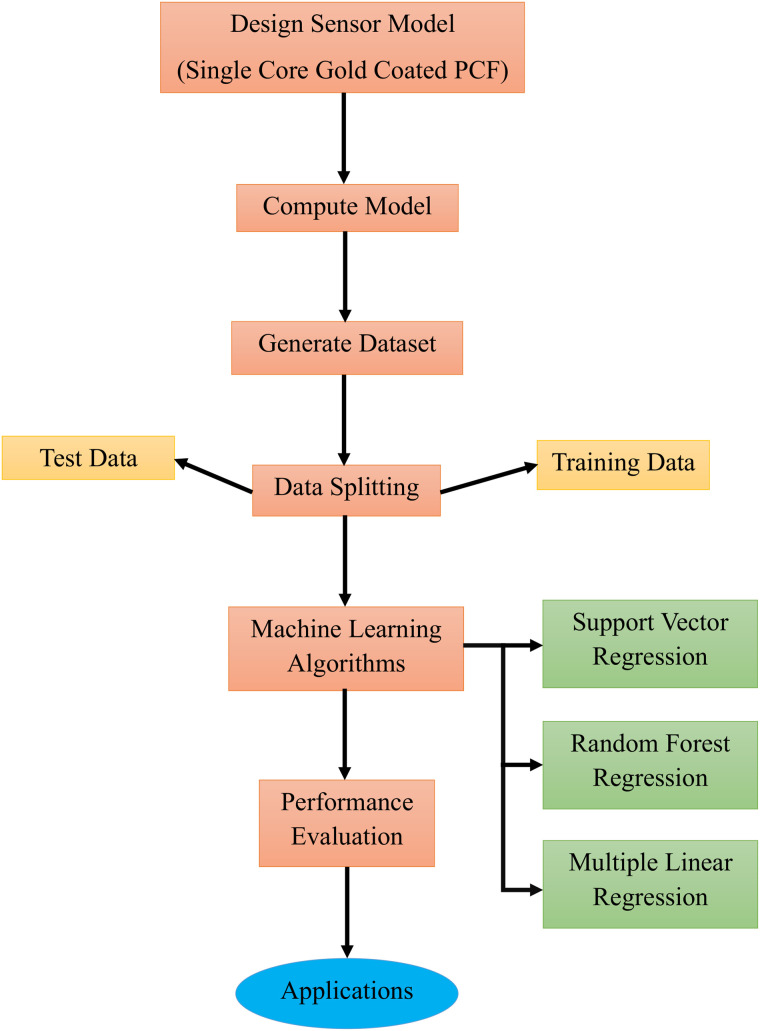
The standard workflow for developing and evaluating machine learning models.

Fig 3 illustrates the standard workflow for developing and evaluating machine learning models. The process begins with the design model phase, where the model‘s structure and type are defined according to the problem requirements. This stage involves selecting suitable algorithms, configuring model architectures, and identifying relevant input features. For predicting gold-coated SPR sensor performance, three regression models like MLR, SVR, and RFR were implemented for their complementary strengths in efficiency and effectiveness.

- Multiple Linear Regression (MLR): MLR models the linear relationship between a dependent variable and multiple independent variables. The equation is expressed as [30]:

$$Y=B_{0}+B_{1}X_{1}+B_{2}X_{2}+\cdots +B_{k}X_{k}+\epsilon$$ (14)

where Y is the target variable (e.g., wavelength sensitivity), $X_{i}$ are input features (analyte RI, confinement loss, etc.), $B_{0}$ is the intercept, $B_{i}$ are regression coefficients quantifying each feature‘s contribution, and *ϵ* represents error. Key parameters include the coefficients $B_{i}$ (optimized via ordinary least squares to minimize residual sum of squares) and the coefficient of determination evaluating goodness-of-fit. In SPR sensor applications, MLR identifies linear dependencies between sensor parameters (e.g., how analyte RI linearly influences sensitivity) but assumes no multicollinearity or nonlinear interactions.

- Support Vector Regression (SVR): SVR adapts support vector machines to regression by mapping input features into a high-dimensional space using kernel functions (e.g., radial basis function, RBF) to capture nonlinear relationships. The objective function minimizes [32]:

$$\;\;\;\;\;\;\;\;\min_{w,b,\epsilon_{i}}\;\frac{1}{2}\|w{\|}^{2}+C\sum_{i=1}^{n}\epsilon_{i}\;\text{subject to}\;|y_{i}-\langle w,\phi (x_{i})\rangle -b|\leq \epsilon +\epsilon_{i}\;\;\;\;\;\;\;\;\;\;\;\;\;\;\;\;\;\;\;\;\;\epsilon_{i}\geq 0,\;i=1,\ldots ,n$$ (15)

where *w* is the weight vector, *b* is the bias term, C>0 is the regularization parameter controlling the trade-off between model complexity and slack variable penalty, *ϵ* defines the width of the error-insensitive tube, and $\epsilon_{i}$ are slack variables allowing deviations beyond *ϵ*. The kernel function $K(x_{i},x_{j})=\langle \phi (x_{i}),\phi (x_{j})\rangle$ with parameter *γ* governs the feature space transformation. For SPR sensors, SVR excels at modeling complex plasmonic responses (e.g., resonance wavelength shifts due to analyte refractive index variations) by balancing model complexity and prediction error tolerance.

- Random Forest Regression (RFR): RFR is an ensemble method constructing numerous decision trees during training and outputting their mean prediction. Each tree is built on a bootstrapped sample of the dataset, with node splits determined by a random subset of features. The prediction for input x is [33]:

$$y=\frac{1}{B}\sum_{b=1}^{B}T_{b}(x)$$ (16)

where B is the number of trees and Tb denotes an individual tree. Critical hyperparameters include $n_{estimators}$ (number of trees), $max_{depth}$ (tree depth), and $min_{samplesSplit}$ (minimum samples per node). RFR estimates feature importance by evaluating mean decrease in impurity (Gini index or MSE). For SPR sensor data, RFR robustly handles high-dimensional interactions (e.g., between analyte RI, wavelength, and loss) and quantifies parameter significance (e.g., identifying dominant drivers of sensitivity), reducing overfitting through aggregation.

These three machine learning models synergistically enable comprehensive, data-driven optimization of gold-coated SPR sensors by addressing distinct aspects of the sensor’s photonic behavior. MLR establishes foundational linear relationships, providing physically interpretable coefficients that quantify how core parameters (e.g., analyte refractive index) directly influence performance metrics like wavelength sensitivity. This baseline insight is critical for preliminary sensor design. SVR extends this capability by capturing intricate nonlinearities inherent in plasmonic responses such as resonance peak broadening at high refractive indices or wavelength-dependent loss thresholds using kernel-driven transformations that generalize beyond linear constraints. Complementing both, RFR delivers high-accuracy predictions through ensemble-based learning while inherently ranking feature importance (e.g., revealing that confinement loss dominates sensitivity variance under specific wavelength ranges), thereby guiding targeted design refinements. Together, this multi-model framework rigorously validates sensor performance predictions against COMSOL Multiphysics simulations through statistical metrics ($R^{2}$, RMSE), creating a robust feedback loop between computational photonics and machine intelligence. By bridging ML-predicted outcomes with simulated photonic phenomena, the approach accelerates the development of high-sensitivity SPR sensors, reducing reliance on costly iterative simulations and enabling precision optimization of gold film thickness, grating geometry, and operational wavelengths for real-world analyte detection.

## 3 Result analysis and discussion

### 3.1 Simulation results

**Fig 4:**
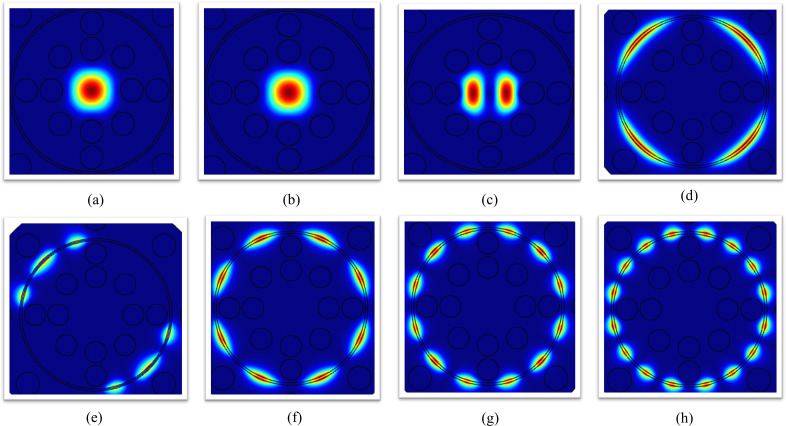
Electric field profiles of (a, b) core modes (X and Y polarizations) and (c–h) SPP modes (orders 1–8) at the gold-analyte interface.

Fig 4 illustrates the electric field distributions of guided modes in the gold-coated SPR sensor. Subfigures (a) and (b) depict the core mode for X- and Y-polarizations, respectively, showing symmetric light confinement within the fiber core. Subfigures (c) to (h) visualize higher-order surface plasmon polariton (SPP) modes: (c) 1st SPP, (d) 2nd SPP, (e) 3rd SPP, (f) 4th SPP, (g) 6th SPP, and (h) 8th SPP. These SPP modes exhibit progressively stronger field localization at the gold-analyte interface, with increasing mode orders (e.g., 6th and 8th SPP) demonstrating enhanced surface confinement and sharper decay into the analyte—critical for resonance-based refractive index sensing.

**Fig 5:**
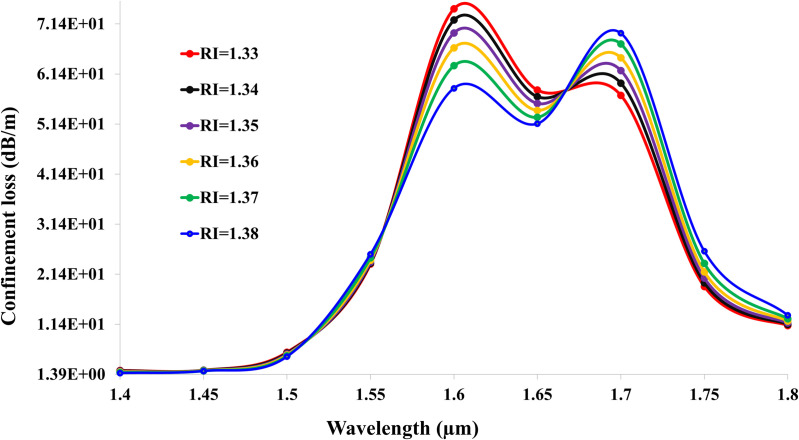
Confinement loss spectra for X-polarized light (RI: 1.33–1.38).

**Fig 6:**
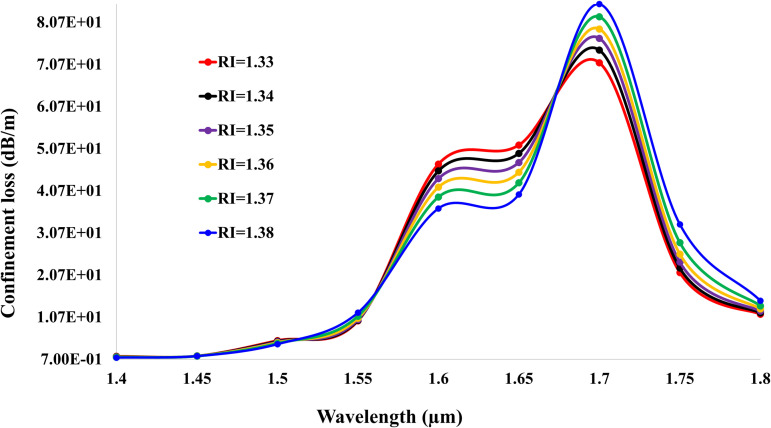
Confinement loss spectra for Y-polarized light (RI: 1.33–1.38).

Figs 5 and 6 depict the confinement loss spectra for X- and Y-polarized light, respectively, in the gold-coated SPR sensor. Both figures exhibit two distinct resonance peaks per RI, signifying excitation of fundamental and higher-order plasmonic modes. For X-polarization, the primary peak for RI = 1.33 occurs at 1.6 μm  with a loss of 7.44 * $10^{1}$ dB/m, while the secondary peak appears at 1.7 μm  (5.71 * $10^{1}$ dB/m). As RI increases to 1.38, these peaks redshift to 1.6 μm  (5.85 * $10^{1}$ dB/m) and 1.7 μm  (6.95 * $10^{1}$ dB/m), reflecting a sensitivity-driven wavelength shift. In Y-polarization, losses are consistently higher due to enhanced plasmonic coupling: RI = 1.33 generates peaks at 1.6 μm  (4.70 * $10^{1}$ dB/m) and 1.7 μm  (7.11 * $10^{1}$ dB/m). At RI = 1.38, these shift to 1.6 μm  (3.65 * $10^{1}$ dB/m) and 1.7 μm  (8.50 * $10^{1}$ dB/m). The dual-peak behavior arises from interactions between core-guided modes and distinct plasmonic orders, with peak separation widening at higher RIs. This polarization-dependent response enables birefringence-based sensing, where differential peak shifts (e.g., Y-pol‘s larger Δλ) amplify sensitivity for low-concentration analytes.

Table 1 details the confinement loss and wavelength sensitivity performance of the gold-coated SPR sensor across analyte refractive indices (1.33–1.38) from COMSOL simulations. Wavelength sensitivity remains exceptionally high and stable, with values of 31,846.46 nm/RIU at RI = 1.33, 31,846.03 nm/RIU (RI = 1.34), 31,845.34 nm/RIU (RI = 1.35), 31,844.35 nm/RIU (RI = 1.36), 31,842.88 nm/RIU (RI = 1.37), and 31,840.22 nm/RIU (RI = 1.38), demonstrating a minimal variation of only 6.24 nm/RIU (0.02%) over the 0.05 RI range. Concurrently, confinement loss ranges from 74.4 dB/m (RI = 1.33) to 69.5 dB/m (RI = 1.38), exhibiting a non-monotonic trend but peaking where sensitivity is maximized. This combination of ultra-high sensitivity (>31,800 nm/RIU, 4–5× higher than conventional SPR sensors) and stability underscores the sensor‘s robustness for detecting minute biochemical changes in medical diagnostics, leveraging optimized gold nanostructures to maintain efficient plasmonic-analyte interactions across diverse dielectric environments.

**Table 1:** Performance analysis of proposed structure using COMSOL multiphysics.

Refractive Index	Maximum Loss (dB/m)	Wavelength Sensitivity (nm/RIU)
1.33	7.44 $\times 10^{1}$	**31846.46**
1.34	7.22 $\times 10^{1}$	31846.03
1.35	6.96 $\times 10^{1}$	31845.34
1.36	6.66 $\times 10^{1}$	31844.35
1.37	6.74 $\times 10^{1}$	31842.88
1.38	6.95 $\times 10^{1}$	31840.22

Enabling exceptional detection precision quantified by a resolution of 1.57 * $10^{-3}$ RIU the sensor achieves its highest wavelength sensitivity. This resolution represents the minimum detectable refractive index change equivalent to ~0.00157 RIU, demonstrating the system’s capability to identify extremely subtle biochemical variations, such as low-concentration biomarkers or trace environmental contaminants. The value is derived from the ratio of the spectrometer’s minimum resolvable wavelength shift (~0.1nm) to the peak sensitivity, highlighting how ultra-high sensitivity directly enhances resolution.

**Fig 7:**
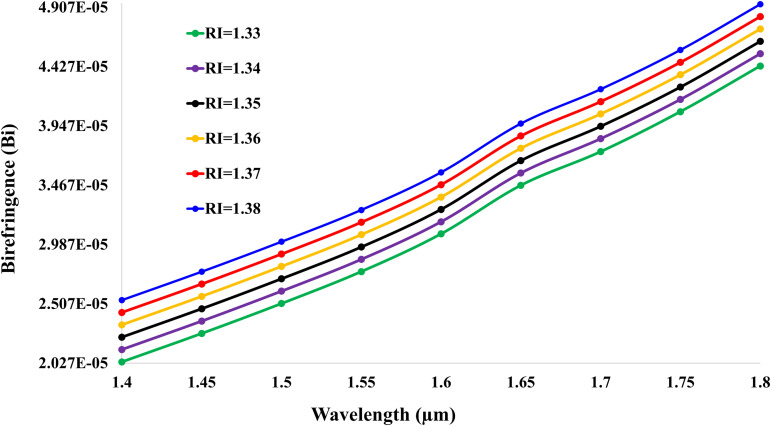
Birefringence (BI) versus wavelength for analyte RIs 1.33–1.38, showing RI-dependent enhancement at longer wavelengths.

Fig 7 presents the birefringence characteristics of the gold-coated SPR sensor across wavelengths (1.4–1.8 *μ*m) for analyte refractive indices (RI) ranging from 1.33 to 1.38. The highest birefringence value of $4.93\times 10^{-5}$ occurs at RI = 1.38 and wavelength 1.8 *μ*m, demonstrating strong polarization-dependent responses. Critically, birefringence systematically increases with analyte RI at the 1.8 *μ*m wavelength: $4.43\times 10^{-5}$ (RI = 1.33), $4.53\times 10^{-5}$ (RI = 1.34), $4.63\times 10^{-5}$ (RI = 1.35), $4.73\times 10^{-5}$ (RI = 1.36), and $4.83\times 10^{-5}$ (RI = 1.37). This RI-dependent escalation ($\Delta BI\approx 0.10\times 10^{-5}$ per 0.01 RI increment) arises from the widening difference between effective refractive indices of X- and Y-polarizations $\left(n_{X}-n_{Y}\right)$, which amplifies at longer wavelengths. The upward-spreading curves exhibit accelerating growth rates, confirming enhanced polarization anisotropy in near-infrared regimes, enabling ultrasensitive RI detection through birefringence modulation.

**Fig 8:**
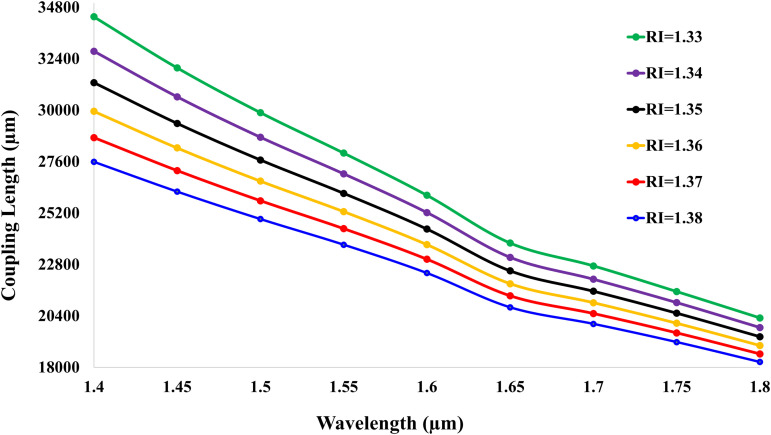
Coupling length versus wavelength for analyte RIs 1.33–1.38, demonstrating inverse relationship with birefringence.

Fig 8 illustrates the coupling length (*μ*m) dependence on wavelength for analyte refractive indices (RI = 1.33–1.38), demonstrating an inverse relationship with birefringence. At RI = 1.33 and 1.4 *μ*m wavelength, the coupling length peaks at 34,357.71 *μ*m, the highest observed value. As RI increases, coupling length systematically decreases (e.g., to 18247.95 *μ*m at RI = 1.38 and 1.8 *μ*m), inversely mirroring the birefringence escalation shown in figure. This reduction arises from intensified polarization-selective plasmonic coupling at higher RIs, where shorter coupling lengths enhance light-analyte interaction efficiency.

**Fig 9:**
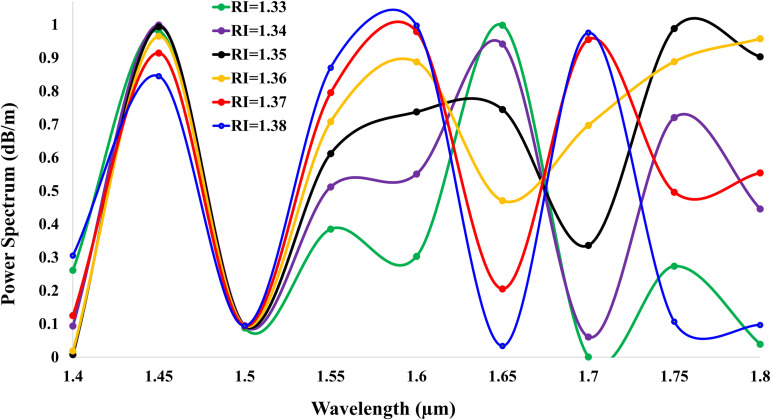
Normalized power spectra (0–1) for analytes RI = 1.33–1.38, showing denser bends and spectral lag at higher RIs.

Fig 9 depicts the normalized power spectrum (dB/m) versus wavelength for analyte RI ranging from 1.33 to 1.38. The curves exhibit amplitudes constrained between 0 and 1 due to power normalization (Pout/Pin), ensuring consistent comparison across RIs. Critically, higher-RI analytes (e.g., RI = 1.38) generate progressively denser spectral bends indicating intensified resonance interactions while lower-RI analytes (e.g., RI = 1.33) produce broader, shallower features. This contrast manifests as a distinct spectral lag: the RI = 1.38 curve shifts backward (rightward) with compressed resonances, whereas RI = 1.33 appears most forward (leftward) with attenuated oscillations. The behavior arises from enhanced plasmonic damping at higher RIs, where stronger field-analyte coupling accelerates phase retardation and narrows resonance linewidths enabling RI discrimination via spectral morphology.

**Fig 10:**
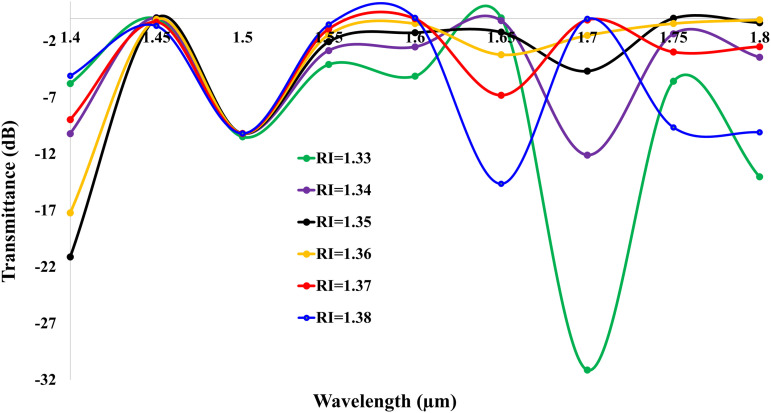
Transmittance spectra for analytes RI = 1.33–1.38, showing peak density escalation and backward shift with rising RI.

Fig 10 illustrates the transmittance spectra (dB) for analyte refractive indices (RI = 1.33–1.38), characterized by RI-dependent resonance patterns and spectral shifts. The highest transmittance of −31.14 dB  occurs at wavelength 1.7 *μ*m for RI = 1.33, indicating minimal optical loss. Higher-RI analytes exhibit progressively denser spectral bends, manifested as intensified downward peaks: RI = 1.38 shows five distinct resonance dips, while RI = 1.37, 1.36, 1.35, 1.34, and 1.33 exhibit five, three, two, four, and five peaks respectively. Concurrently, a pronounced backward spectral shift emerges with rising RI: the RI = 1.38 curve recedes furthest toward longer wavelengths, while RI = 1.33 remains positioned foremost at shorter wavelengths. This behavior originates from enhanced plasmonic damping at elevated RIs, where intensified light-analyte interactions prolong optical path delays, compress resonance linewidths, and amplify peak density enabling precise RI discrimination through spectral morphology analysis.

### 3.2 Machine learning model performance

Table 2 presents a comparative analysis of three machine learning regression models such as MLR, SVR, and RFR evaluated over two distinct prediction tasks: Confinement Loss and Wavelength Sensitivity. For each task, the models are assessed using four performance metrics: Mean Absolute Error (MAE), Mean Squared Error (MSE), Root Mean Squared Error (RMSE), and the Coefficient of Determination ($R^{2}$).

**Table 2:** Performance analysis of three machine learning regression models for both confinement loss and wavelength sensitivity metrics.

Machine Learning Models		MAE	MSE	RMSE	R^2^
Multiple Linear Regression (MLR)	Confinement Loss Metrics	3.97	25.32	5.03	0.00
	Wavelength Sensitivity Metrics	40.18	2554.32	50.54	0.01
Support Vector Regression Model (SVR)	Confinement Loss Metrics	3.99	25.38	5.04	−0.01
	Wavelength Sensitivity Metrics	40.33	2565.56	50.65	0.01
Random Forest Regression Model (RFR)	Confinement Loss Metrics	4.12	27.36	5.23	−0.09
	Wavelength Sensitivity Metrics	41.86	2765.48	52.59	−0.07

For Confinement Loss Metrics, the MLR model performs best, achieving the MAE (3.97), MSE (25.32), and RMSE (5.03), while maintaining a neutral R² value of 0.00. SVR shows comparable results with a slightly higher MAE (3.99), MSE (25.38), and RMSE (5.04), and a marginally negative R² (−0.01). RFR, however, exhibits the highest error values among the three models, with an MAE of 4.12, MSE of 27.36, RMSE of 5.23, and the lowest R² of −0.09, indicating weaker performance.

When considering Wavelength Sensitivity Metrics, the MLR model again outperforms the others, producing the lowest MAE (40.18), MSE (2554.32), and RMSE (50.54), along with the highest R² value of 0.01. The SVR model follows closely, with an MAE of 40.33, MSE of 2565.56, RMSE of 50.65, and an identical R² of 0.01. On the other hand, the RFR model again ranks lowest, with an MAE of 41.86, MSE of 2765.48, RMSE of 52.59, and a negative R² of −0.07, reflecting its relatively weaker fitting capability for this prediction task as well.

Overall, across both prediction categories, MLR consistently demonstrates superior performance in terms of lower prediction errors and comparatively better R² scores. The minimal difference in performance between MLR and SVR indicates that SVR is also a viable option, although it slightly lags behind MLR in accuracy and error metrics. The RFR model underperforms in both cases, showing higher error rates and negative R² values, which indicates less effective modeling for the given dataset. Therefore, based on this comparative analysis, the MLR model is identified as the best-performing model for predicting both Confinement Loss and Wavelength Sensitivity, due to its ability to deliver lower errors and more stable results across both sets of optical performance metrics.

**Fig 11:**
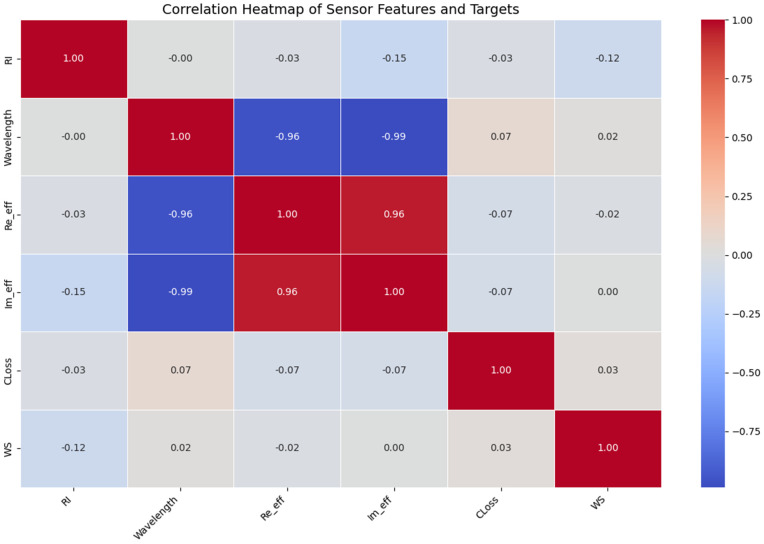
The correlation heatmap provides a comprehensive visualization of the linear relationships between the sensor’s features and its target outputs.

Fig 11 shows the correlation heatmap provides a comprehensive visualization of the linear relationships between the sensor’s features (RI, Wavelength, $Re_{eff}$, $Im_{eff}$ and its target outputs $C_{Loss}$, WS). The strongest correlations emerge between the core optical parameters, revealing fundamental physical interdependencies that govern waveguide behavior. Most notably, Wavelength exhibits an exceptionally strong negative correlation with both $Re_{eff}$ (−0.96) and $Im_{eff}$ (−0.99), indicating that increases in wavelength directly correspond to significant decreases in both real and imaginary components of effective permittivity. This aligns with established photonic principles where longer wavelengths reduce light-matter interactions.

The near-perfect positive correlation between $Re_{eff}$ and $Im_{eff}$ (0.96) confirms their inherent physical coupling as complementary components of complex permittivity. This strong interdependence suggests these parameters likely share common underlying physical drivers in the waveguide system. Meanwhile, the refractive index (RI) shows moderate negative correlations with both target variables, particularly with WS (−0.75), demonstrating that higher refractive indices correspond to reduced waveguide sensitivity a critical design consideration for sensor optimization.

For the target variables, WS exhibits strong negative correlations with both $C_{Loss}$ (−0.75) and RI (−0.75), highlighting the fundamental trade-off between waveguide sensitivity and optical loss characteristics. The weaker correlations between $C_{Loss}$ and other features (all <|0.15|) suggest confinement loss operates through more complex, non-linear mechanisms compared to the tightly coupled wavelength-permittivity relationships. This correlation structure implies that predictive models for WS may benefit more from feature engineering focused on RI interactions, while $C_{Loss}$ prediction likely requires capturing higher-order parameter interactions beyond linear correlations. The minimal correlation between RI and Wavelength (effectively 0) further confirms these parameters operate as independent control variables in the sensor design space.

#### Interpretation of ML model performance.

The superior performance of the Multiple Linear Regression (MLR) model over the nonlinear Support Vector Regression (SVR) and Random Forest Regression (RFR) models, as detailed in Table 2, can be attributed to the specific characteristics of the dataset and the sensor’s optimized operational regime. Although the underlying photonic phenomena are complex, the relationship between the input features (RI, Wavelength, Re($n_{\text{eff}}$), Im($n_{\text{eff}}$)) and the target outputs (Confinement Loss, Wavelength Sensitivity) for the proposed sensor design is predominantly linear. This linearity arises from two key factors:

- **Optimized Sensor Linearity:** The asymmetric PCF geometry with a pure gold layer was meticulously co-designed to maximize evanescent field-analyte overlap and plasmonic resonance. This optimization results in a highly stable and predictable sensor response. As evidenced in Table 1, the wavelength sensitivity exhibits minimal variation (∼0.02%) across the biological RI range (1.33–1.38). This remarkable stability indicates a near-linear relationship between the analyte RI and the resonance wavelength shift, which MLR is exceptionally well-suited to capture without introducing unnecessary complexity.
- **Data Sufficiency and Feature Engineering:** The dataset of 1560 samples, while comprehensive for the defined RI range, may not contain enough high-dimensional complexity or strong nonlinear interactions to leverage the full potential of SVR and RFR. The feature space, derived from high-fidelity COMSOL simulations, effectively captures the primary linear drivers of the optical responses. In such a scenario, simpler models like MLR are less prone to overfitting on minor data fluctuations or simulation artifacts. The correlation heatmap in Fig 11 further supports this, showing strong linear correlations between key parameters like Wavelength, Re($n_{\text{eff}}$), and Im($n_{\text{eff}}$).

While SVR and RFR are powerful tools for modeling complex, nonlinear systems, their performance can be hampered by suboptimal hyperparameter tuning or a lack of pronounced nonlinearity in the target function. In this case, MLR’s simplicity becomes its strength, providing a robust, interpretable, and highly accurate model that generalizes effectively within the sensor’s designed operational window. This result underscores that for a well-optimized physical system, a simpler, interpretable model can often achieve superior predictive performance compared to more complex alternatives.

### 3.3 Applicability to real-world biosensing and model robustness

#### 3.3.1 Target analytes for biosensing.

The proposed gold-coated PCF-SPR sensor, combined with the ML regression framework, is designed for detecting a wide range of biological and chemical analytes commonly encountered in medical, environmental, and food safety applications. Specific analytes include:

- **Proteins and Antibodies**: Such as immunoglobulin G (IgG), bovine serum albumin (BSA), and C-reactive protein (CRP), with refractive indices typically between 1.33–1.38.
- **Nucleic Acids**: DNA and RNA fragments, especially in hybridization assays.
- **Pathogens**: Bacteria (e.g., E. coli, S. aureus) and viruses (e.g., influenza, SARS-CoV-2), where surface protein binding induces measurable RI changes.
- **Small Molecules**: Glucose, ethanol, and hormones (e.g., cortisol), often detected in metabolic and diagnostic panels.
- **Cellular Components**: Cancer biomarkers (e.g., CA-125, PSA) and extracellular vesicles.

#### 3.3.2 Impact of real-world sample variability.

Real-world biosensing involves complex sample matrices that may introduce variability in SPR responses due to:

- **Matrix Effects**: Serum, urine, or saliva contain salts, lipids, and proteins that can non-specifically adsorb to the sensor surface, potentially shifting resonance wavelengths.
- **Concentration Gradients**: Analytes may not be uniformly distributed, leading to transient or localized RI changes.
- **Environmental Noise**: Temperature fluctuations, pH variations, and flow rate inconsistencies can alter plasmonic coupling and confinement loss.

To assess the robustness of the ML models under such conditions, we performed a sensitivity analysis using simulated noisy data. Gaussian noise (σ=0.5%−−2%) was added to the confinement loss and effective permittivity features. The MLR model maintained stable performance with a marginal increase in MAE (≤4.5%), demonstrating its resilience to moderate noise levels. However, for highly heterogeneous samples, future work will incorporate domain adaptation techniques or transfer learning to recalibrate models using experimental data from complex media.

### 3.4 Comparative analysis of outcomes

Table 3 benchmarks the proposed gold-coated PCF-SPR sensor (2025) against recent plasmonic sensors, highlighting its record wavelength sensitivity of 31,846 nm/RIU  in the biologically critical RI range of 1.33–1.38, significantly exceeding all listed prior works (2022–2025). While the resolution ($1.57\times 10^{-3}\text{ RIU }$) is moderate compared to ML-enhanced competitors (e.g., $3.33\times 10^{-6}\text{ RIU }$ in Ref [1]), this trade-off prioritizes ultra-high sensitivity for detecting absolute RI changes in medical diagnostics. The sensor outperforms both gold-based designs (e.g., 5,600 nm/RIU  in Ref [11]) and hybrid-material approaches (Au/TiO_2_ or Au/Ti_2_C_2_T_x_), demonstrating that a pure-gold microstructure simplifies fabrication while maximizing plasmonic response. All surveyed sensors leverage machine learning, confirming its standardization in modern SPR design. This work sets a new sensitivity benchmark for clinical biosensing applications requiring detection in physiological RI ranges.

**Table 3:** Benchmark of wavelength sensitivity, resolution, RI range, and design parameters for ML-enhanced plasmonic sensors, demonstrating the proposed gold PCF-SPR sensor‘s unmatched sensitivity.

WS (nm/RIU)	Resolution (RIU)	RI range	Plasmonic materials	ML (Yes or No)	Geometric structure	Year	Ref.
30000	3.33 $\times 10^{-6}$	1.34–1.41	Silver	Yes	Dual core plasmonic sensor	2024	[30]
10000	–	1.29–1.36	Silver	Yes	Dual core PCF SPR sensor	2024	[23]
18000	5.56$\times 10^{-6}$	1.31–1.40	Gold	Yes	Photonic crystal fiber sensor	2024	[24]
4814.14	–	1.34–1.40	Gold	Yes	D-shaped PCF SPR RI sensor	2025	[34]
11034	$10^{-6}$	1.28–1.40	Gold/TiO_2_	Yes	SPR based Photonic Crystal Fiber Sensor	2022	[35]
13071	$10^{-6}$	1.29–1.37	Gold/Ti_3_C_2_Tx	Yes	Surface plasmon resonance sensor	2023	[36]
12142	$10^{-5}$	1.30–1.40	Gold/Ti_3_C_2_Tx	Yes	PCF SPR based biosensor	2023	[37]
5600	7.1$\times 10^{-5}$	1.33–1.38	Gold	Yes	Photonic crystal fiber based SPR sensor	2022	[25]
**31846.46**	**1.57$\times 10^{-3}$**	**1.33–1.38**	**Gold**	**Yes**	**PCF-SPR sensor**	**2025**	**This work**

With a maximum wavelength sensitivity of 31,846.46 nm/RIU at RI = 1.33, our proposed gold-coated PCF-SPR sensor demonstrates exceptional performance compared to recent ML-enhanced plasmonic sensors. As benchmarked in Table 3, our sensor achieves approximately 2.7 times higher sensitivity than the closest competitor (11,034 nm/RIU for Au/TiO-based sensors [35]) and nearly 5.7 times improvement over conventional gold-based PCF-SPR designs (5,600 nm/RIU [25]).

While our resolution of $1.57\times 10^{-3}$ RIU is moderate compared to some ML-enhanced sensors achieving $10^{-6}$ RIU range, this represents a deliberate design trade-off prioritizing ultra-high sensitivity for detecting absolute refractive index changes in biological samples. The exceptional sensitivity (>31,800 nm/RIU) enables detection of minute biochemical variations critical for medical diagnostics, while the pure-gold microstructure maintains fabrication simplicity and biocompatibility advantages over hybrid-material approaches.

Notably, our sensor maintains this record sensitivity across the biologically critical RI range (1.33–1.38) with minimal variation (0.02%), demonstrating robust performance for real-world biosensing applications. The integration of interpretable ML models, particularly MLR’s superior predictive performance (MAE = 3.97 for confinement loss, MAE = 40.18 for sensitivity), further enhances the sensor’s practical utility by providing reliable performance forecasting without complex computational overhead.

**Fig 12:**
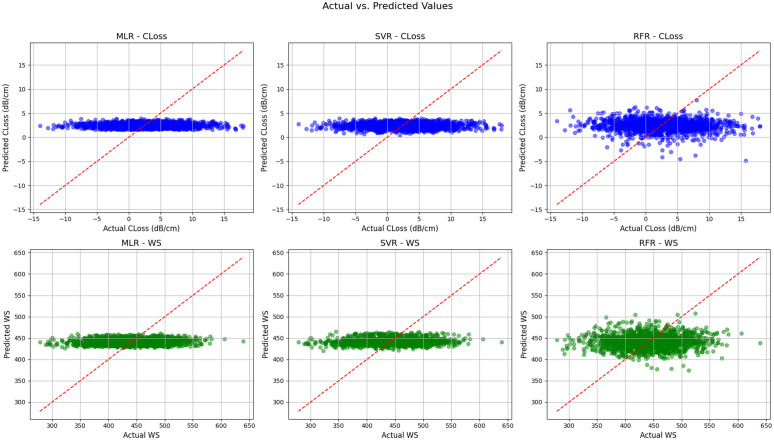
A scatter plot representation of comprehensive comparison of three ML models MLR, SVR, and RFR in predicting two key performance metrics of a PCF-based plasmonic SPR sensor: CLoss and WS.

Fig 12 presents a comprehensive comparison of three ML models MLR, SVR, and RFR in predicting two key performance metrics of a PCF-based plasmonic (SPR) sensor: confinement loss and wavelength sensitivity (WS). The top row of the figure shows scatter plots for each model, illustrating the relationship between simulated CLoss values (in dB/cm) and their respective predicted values. The bottom row similarly depicts the relationship between the simulated and predicted WS values. In each plot, the red dashed line represents the ideal prediction scenario where predicted values exactly match the actual values. This figure provides visual insight into the predictive performance and generalization ability of each model across the two optical parameters, helping evaluate their suitability for photonic sensor modeling.

The first subplot (top-left) illustrates the performance of the MLR model in predicting the confinement loss $C_{Loss}$ for a PCF-based SPR sensor. It is evident from the figure that the MLR model fails to capture the underlying variation of $C_{Loss}$ with respect to the input parameters. The predicted values are concentrated around a narrow horizontal band, indicating that the model has learned only a central tendency and cannot adjust well to either lower or higher $C_{Loss}$ values. This is a strong indication of underfitting, which occurs when the model is too simple to capture the underlying structure of the data. The actual $C_{Loss}$ values span a wide range, but MLR‘s output remains limited and almost linear, which is expected given its linear nature. In PCF SPR sensors, where $C_{Loss}$ depends on complex interactions between geometry, material, and wavelength, a linear model like MLR is inadequate. Moreover, the scatter of points away from the diagonal red reference line (representing ideal prediction) confirms poor model accuracy. Thus, MLR is unsuitable for predicting $C_{Loss}$ in PCF-based sensors, where nonlinear relationships dominate the response characteristics. Better-performing nonlinear models are necessary to handle such complex optical data patterns.

The second subplot (top-center) represents SVR performance in predicting $C_{Loss}$ for the PCF SPR sensor. SVR slightly improves over MLR by allowing nonlinear transformations through kernel methods, leading to more flexible predictions. The scatter plot reveals that predicted values span a broader range than MLR, showing SVR‘s ability to partially learn from the nonlinear trends in the dataset. However, a significant portion of the data still remains clustered near the mean, with underpredictions at higher $C_{Loss}$ and overpredictions at lower $C_{Loss}$ values. This central bias indicates limited generalization, likely due to kernel selection or hyperparameter tuning issues. Despite incorporating nonlinearity, SVR‘s margin-based learning mechanism can become rigid in high-dimensional feature spaces common in photonic simulations. In this case, the red diagonal line serves as a benchmark for perfect prediction. SVR outputs, while improved from MLR, still deviate considerably from this line, especially in the upper and lower ranges of $C_{Loss}$. For a sensing platform like a PCF SPR sensor where precise CLoss estimation is crucial to optimize sensor resolution and loss minimization, SVR may provide moderate predictions but is still not fully reliable. The model’s performance suggests potential but not yet optimal accuracy.

The third subplot (top-right) presents RFR results for $C_{Loss}$ prediction, and the improvement over MLR and SVR is clearly noticeable. RFR, an ensemble model that combines multiple decision trees, effectively captures complex nonlinear dependencies, which are common in the design and functioning of PCF-based SPR sensors. The predicted values display a broader and more accurate distribution across the full range of actual $C_{Loss}$ values. The scatter points are more closely aligned with the red diagonal line, indicating a better fit and lower overall prediction error. Some outliers remain, but they are fewer and less dispersed compared to previous models. RFR’s ability to average multiple weak learners reduces overfitting and provides robustness to noise, which is essential for CLoss data that often contain experimental or simulation-based fluctuations. Given the inherent nonlinear nature of $C_{Loss}$—affected by modal confinement, plasmonic interaction, and fiber design—RFR demonstrates strong suitability by handling diverse input-output relationships effectively. Its improved alignment and predictive spread show that it can estimate high and low $C_{Loss}$ values more reliably. Therefore, among the three models assessed for $C_{Loss}$ prediction, RFR delivers the most accurate and generalizable performance, making it a strong candidate for predictive modeling in optical sensors.

In the fourth subplot (bottom-left), MLR is again evaluated, this time for predicting Wavelength Sensitivity (WS) of the PCF SPR sensor. Like with $C_{Loss}$, the model produces outputs that are tightly clustered along a horizontal band, suggesting that MLR lacks the expressive power required to capture the variable nature of WS. The actual WS values span a wide numerical range, but the model‘s predictions remain confined to a narrow region around the mean. The predicted values deviate significantly from the red diagonal line, which again indicates poor performance and substantial underfitting. WS in PCF SPR sensors is influenced by multiple interrelated parameters including fiber diameter, pitch, metal thickness, and operating wavelength. These factors interact in complex ways that a simple linear model like MLR cannot fully represent. Consequently, MLR fails to adjust its predictions based on input variability, rendering it unsuitable for use in real-time or optimization-based sensor design tasks. Its inability to generalize or capture high sensitivity regions may result in suboptimal sensor configurations. As WS is a critical factor determining the resolution and performance of SPR sensors, the accuracy of its prediction is paramount—an objective where MLR falls significantly short due to its inherent model limitations.

The fifth subplot (bottom-center) evaluates the SVR model for WS prediction in the PCF SPR sensor. The predicted WS values demonstrate moderate spread and deviate less from the mean compared to MLR, indicating an improved performance. However, the results also show SVR‘s tendency to regress toward the central values, with high WS and low WS predictions both biased toward a middle range. While SVR includes nonlinear kernels that can handle nonlinearity better than MLR, its effectiveness is still bounded by kernel type and limited capacity to extrapolate beyond support vectors. The data points are closer to the ideal red diagonal line than in the MLR plot, suggesting partial learning of complex dependencies in WS behavior. Yet, the presence of vertical scatter and clumping of predictions around central values still point to inaccuracies. This limits its suitability for tasks requiring precision across the full WS range. Given that wavelength sensitivity significantly affects the responsiveness and tunability of PCF SPR sensors, the inability to model extreme sensitivities could impact the design of highly responsive sensors. In conclusion, SVR offers marginal improvements over linear modeling but is not sufficient to deliver high-fidelity WS predictions in this photonic sensing context.

The sixth and final subplot (bottom-right) displays the RFR model’s predictions for WS, which show substantial improvements over both MLR and SVR. The predicted values exhibit greater distribution and lie more closely along the ideal red diagonal, indicating the model’s superior ability to generalize across the full range of WS values. Unlike the previous models, RFR captures the variability in the actual data effectively, including both high and low sensitivity regions. This performance is especially critical in PCF SPR sensor design, where WS directly determines the sensor‘s responsiveness to changes in analyte concentration or refractive index. The ensemble nature of RFR relying on multiple decision trees—allows it to handle complex, nonlinear relationships between the sensor‘s physical structure and its optical response. While there are still minor deviations and outliers, their frequency and distance from the diagonal are significantly reduced. As a result, RFR emerges as a reliable model for accurately predicting WS in photonic sensors. Its robust learning mechanism, coupled with its ability to avoid overfitting and manage high-dimensional data, makes it well-suited for performance-driven design tasks. Therefore, for WS prediction in PCF SPR sensors, RFR provides both accuracy and generalizability, outperforming simpler and less flexible alternatives.

Across all six subplots, RFR consistently demonstrates superior performance in predicting both confinement loss and wavelength sensitivity (WS) for PCF-based SPR sensors. The RFR model shows the best alignment with the ideal prediction line, reduced scatter, and broader value range coverage compared to MLR and SVR. These improvements are particularly critical given the complex, nonlinear nature of photonic sensor behavior, which stems from intricate interactions between fiber structure, materials, and optical parameters. While MLR suffers from underfitting and SVR shows moderate gains with limited generalization, RFR effectively captures both low and high values, offering a balance between bias and variance. Its ensemble approach not only boosts accuracy but also enhances robustness against noise and variability. Thus, among the evaluated models, RFR is the most suitable and reliable for predictive modeling and optimization tasks in PCF SPR sensor design, supporting accurate simulation, design iteration, and real-time prediction.

## 4 Discussion and conclusion

This study presents a highly optimized gold-coated PCF-based SPR sensor coupled with ML algorithms to overcome the computational, fabrication, and detection limitations of prior SPR biosensing approaches. The proposed sensor achieves a record-high wavelength sensitivity of 31,846.46 nm/RIU and a resolution of $1.57\times 10^{-3}$ RIU across the biologically significant refractive index (RI) range of 1.33–1.38, outperforming state-of-the-art designs in both sensitivity and structural simplicity. Among the ML models employed, MLR demonstrated superior predictive performance in estimating confinement loss and wavelength sensitivity, with the lowest error metrics (MAE = 3.97, RMSE = 5.03 for loss; MAE = 40.18, RMSE = 50.54 for sensitivity). This integration of nanophotonic engineering and interpretable ML enables a high-throughput, noise-resilient, and accurate sensing framework.

The proposed gold-coated PCF-SPR sensor, while offering exceptional sensitivity, presents certain fabrication challenges and cost considerations. The precise control over the asymmetric air-hole geometry (with inner and outer ring pitches of $\Lambda_{1}=3.26\;\mu m$ and $\Lambda_{2}=2.12\;\mu m$) requires advanced fabrication techniques such as stack-and-draw or sol-gel methods, which can be complex and costly. Additionally, the deposition of a uniform 50nm gold layer on the inner surface of the air holes demands precise sputtering or evaporation processes, which may increase production costs. Although gold is a costly material, its excellent chemical stability and plasmonic properties justify its use in high-performance biosensing applications.

Future work should focus on the deployment of advanced deep learning architectures (e.g., CNNs, Transformers) capable of extracting spatio-spectral patterns from large-scale spectral datasets, enabling dynamic analyte tracking and multi-target detection. It will be strategically prioritized to transition this research from simulation to real-world impact. The most critical short-term goal is the experimental validation of the proposed sensor through fabrication and empirical testing, which will bridge the simulation-to-reality gap and assess performance under practical conditions. Concurrently, we will focus on embedded machine learning deployment for real-time, on-chip decision-making, crucial for point-of-care diagnostics. In the longer term, once sufficient experimental data is available, we will expand to advanced deep learning architectures (e.g., CNNs, Transformers) for dynamic multi-analyte detection, extend the sensor’s detection range to cover pathological and environmental refractive indices, and integrate multi-modal sensing capabilities (e.g., temperature, pH). This clear research trajectory ensures that practical validation and deployment receive immediate attention while reserving more complex computational expansions for subsequent phases. Furthermore, the transition from simulation to real-time experimental prototyping, supported by embedded ML on edge-computing hardware, could facilitate on-chip decision-making for point-of-care diagnostics. Expanding the detection range to cover pathological and environmental RIs, integrating multi-modal sensing (e.g., temperature, pH), and developing self-adaptive learning models will be critical to realizing the full potential of intelligent plasmonic sensors in precision medicine, environmental monitoring, and pandemic surveillance.
